# Loss and Recovery of *Mgat3* and GnT-III Mediated E-cadherin N-glycosylation Is a Mechanism Involved in Epithelial-Mesenchymal-Epithelial Transitions

**DOI:** 10.1371/journal.pone.0033191

**Published:** 2012-03-13

**Authors:** Salomé S. Pinho, Patrícia Oliveira, Joana Cabral, Sandra Carvalho, David Huntsman, Fátima Gärtner, Raquel Seruca, Celso A. Reis, Carla Oliveira

**Affiliations:** 1 Institute of Molecular Pathology and Immunology of the University of Porto, Porto, Portugal; 2 Institute of Biomedical Sciences of Abel Salazar, University of Porto, Porto, Portugal; 3 British Columbia Cancer Agency, Vancouver, British Columbia, Canada; 4 Medical Faculty, University of Porto, Porto, Portugal; Virginia Commonwealth University, United States of America

## Abstract

**Background:**

N-acetylglucosaminyltransferase-III (GnT-III) is a glycosyltransferase encoded by *Mgat3* that catalyzes the addition of β1,4-bisecting-N-acetylglucosamine on N-glycans. GnT-III has been pointed as a metastases suppressor having varying effects on cell adhesion and migration. We have previously described the existence of a functional feedback loop between E-cadherin expression and GnT-III-mediated glycosylation. The effects of GnT-III-mediated glycosylation on E-cadherin expression and cellular phenotype lead us to evaluate *Mgat3* and GnT-III-glycosylation role during Epithelial-Mesenchymal-Transition (EMT) and the reverted process, Mesenchymal-Epithelial-Transition (MET).

**Methodology/Principal Findings:**

We analyzed the expression profile and genetic mechanism controlling *Mgat3* expression as well as GnT-III-mediated glycosylation, in general and specifically on E-cadherin, during EMT/MET. We found that during EMT, *Mgat3* expression was dramatically decreased and later recovered when cells returned to an epithelial-like phenotype. We further identified that *Mgat3* promoter methylation/demethylation is involved in this expression regulation. The impact of *Mgat3* expression variation, along EMT/MET, leads to a variation in the expression levels of the enzymatic product of GnT-III (bisecting GlcNAc structures), and more importantly, to the specific modification of E-cadherin glycosylation with bisecting GlcNAc structures.

**Conclusions/Significance:**

Altogether, this work identifies for the first time *Mgat3* glycogene expression and GnT-III-mediated glycosylation, specifically on E-cadherin, as a novel and major component of the EMT/MET mechanism signature, supporting its role during EMT/MET.

## Introduction

Glycosylation is considered the most abundant post-translational modification and occurs when oligosaccharides (glycans) are covalently attached to other biomolecules such as proteins or lipids [1]. Glycosylation can have a prominent role in several fundamental biological processes such as embryogenesis, development, growth, differentiation and also in diseases such as cancer [2], [3], [4]. Glycosylation is catalyzed by several glycosyltransferases that act in a step-wise manner. Functional glycomics, in particular, is currently allowing the understanding of the role played by glycosyltransferases and glycans in cell biology and function of organisms [5].

N-acetylglucosaminyltransferase III (GnT-III) is a glycosyltransferase encoded by the *Mgat3* gene that catalyzes the transfer of N-acetylglucosamine (GlcNAc) in a β1,4 linkage to mannose on N-glycans forming a bisecting GlcNAc structure [6]. GnT-III is considered a key glycosyltransferase in N-glycan biosynthetic pathway since the introduction of the bisecting GlcNAc residue precludes further processing and elongation of N-glycans catalyzed by GnT-V, suppressing the formation of β1,6 GlcNAc branching structures [7]. In a tumour context, GnT-III and GnT-V have generally a dual role where GnT-III and its bisecting GlcNAc structures act as metastases suppressors whereas GnT-V and its β1,6 GlcNAc branching structures are associated with increased malignancy and metastasis [8].

The detailed molecular mechanism and the most important molecular targets underlying this antagonistic role of GnT-III and GnT-V in cancer development and progression have recently been addressed [8], [9], [10]. Focusing on the role of GnT-III as an important metastases suppressor, Yoshimura et al. have demonstrated that GnT-III gene transfected into B16 mouse melanoma cells with high metastatic capacity led to a suppression in the formation of β1,6 GlcNAc branching structures catalyzed by GnT-V, together with a significant decrease in lung colonization after mice intravenous administration of GnT-III transfectants [11]. Further on, the same group showed that in those GnT-III transfectants from mouse melanoma cells, E-cadherin was modified with bisecting GlcNAc structures showing a delayed turnover rate and an increased cell-cell adhesion, which might explain the metastatic impairment induced by GnT-III overexpression [12]. The anti-metastatic role of GnT-III was also described by Isaji *et al.*, in a report showing that modification of Integrin α5β1 with bisecting GlcNAc structures, catalyzed by GnT-III, inhibits cell spreading and migration on fibronectin [13]. According with these results, it was proposed that GnT-III prevents tumour metastasis by at least two mechanisms: enhancement of cell-cell adhesion (through E-cadherin glycosylation) and down-regulation of cell-ECM adhesion (through Integrin glycosylation) [9]. Moreover, it was also demonstrated that the addition of bisecting GlcNAc structures catalyzed by GnT-III to mammary tumour cell glycoprotein receptors inhibits growth factor signalling reducing growth and retarding mammary tumour progression [14]. Recently, our group has proposed a mechanistic model where E-cadherin expression regulates the *Mgat3* gene transcription leading to increased expression of GnT-III enzyme that in turn glycosylates E-cadherin by adding bisecting GlcNAc structures [15], which promotes its stability and function at the cell membrane (unpublished results). Interestingly, when this functional feed-back loop between E-cadherin and GnT-III is disturbed, by performing GnT-III knockdown, we observed a dramatic alteration in the cell morphology with formation of filopodia and lamellipodia extensions, together with a delocalization of E-cadherin from the cell membrane into the cytoplasm [15].

E-cadherin is a well-accepted marker of phenotypic plasticity [16], and is the central target and the most common endpoint of Epithelial to Mesenchymal Transition (EMT) signalling pathways [17]. This is valid for EMT, but also to the apparent reverse process known as Mesenchymal to Epithelial Transition (MET), that occurs during embryonic development, tissue regeneration, wound-healing and thought to occur in cancer initiation/progression [18], [19], [20]. The most commonly used epithelial molecular markers include E-cadherin, occludin, cytokeratins, whereas N-cadherin and vimentin are classically considered mesenchymal markers [21]. EMT is also characterized by increased production of transcription factors such as Snail, Slug, Twist, ZEB1, ZEB2, and/or E47, some of which also known as EMT inducers. During EMT/MET, cells alternate between pure epithelial and mesenchymal phenotypes characterized by modifications in cell adhesion, polarity, migration and cell shape [16], [17]. This EMT/MET associated cell plasticity is mirrored by many features induced by overexpression of GnT-III (associated with epithelial phenotype) and GnT-V (associated with mesenchymal features) [15], [22].

Altogether, some reports as well as our previous results show an effect of GnT-III mediated glycosylation on cellular phenotype and E-cadherin cellular expression [10], [15], [23]. We therefore hypothesize that during EMT/MET, E-cadherin could be post-translationally regulated by GnT-III-mediated glycosylation.

In the present study, we analyzed the expression profile and the genetic mechanism controlling *Mgat3* gene expression as well as the GnT-III-induced glycosylation levels, in general and specifically on E-cadherin, during EMT/MET. We found that during EMT, *Mgat3* gene suffered a dramatic decrease in expression that was significantly recovered when cells re-acquired an epithelial-like phenotype (MET). E-cadherin was specifically targeted and regulated by GnT-III mediated glycosylation during EMT/MET. We propose here, for the first time, *Mgat3*/GnT-III mediated glycosylation as a novel and major mechanism of E-cadherin regulation during EMT/MET.

## Results

### TGF-β1 induces EMT in *EpH-4* non-tumourigenic mammary epithelial cells and TGF-β1 removal initiates the MET program

**Figure 1:**
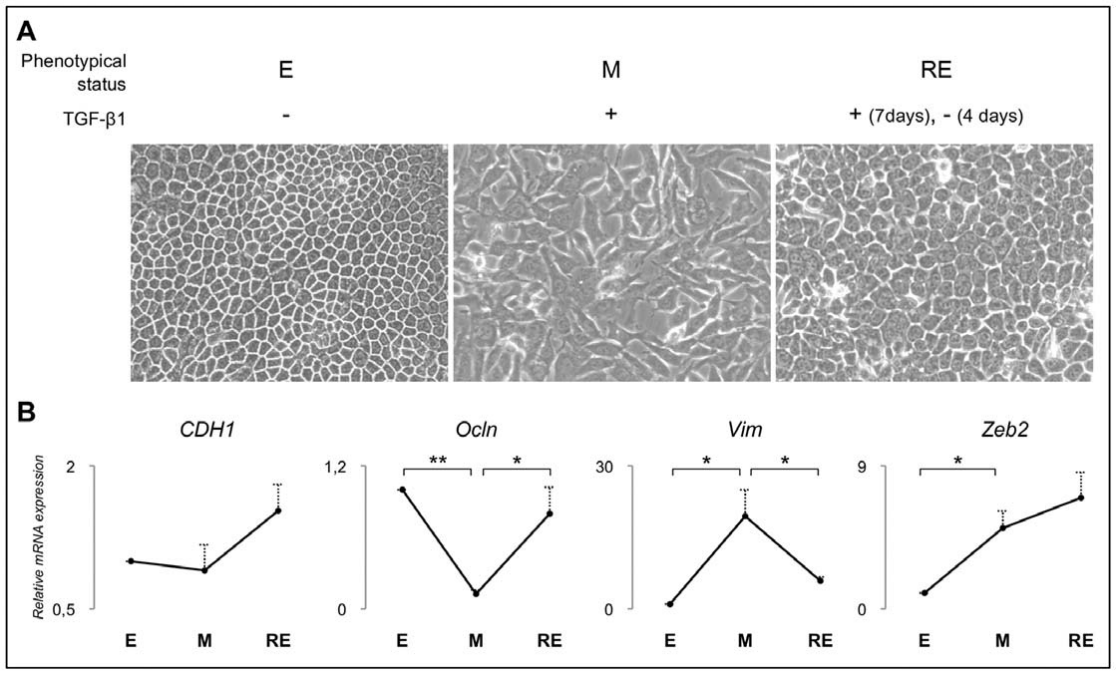
EMT/MET model validation. Panel A shows bright field microscopy still images of the *EpH-4* cell line (400×) during EMT and MET induction. E stands for epithelial cells, M stands for mesenchymal cells, and RE stands for reverted epithelial cells. E and RE cells display a cuboid shape associated with the epithelial phenotype while M cells exhibit a fibroblastic morphology associated with the mesenchymal phenotype. Panel B illustrates the quantification of *CDH1*, *Ocln*, *Vim* and *Zeb2* relative mRNA expression (*n* = 3 biological replicas). Data was normalized for results obtained for E cells. Single asterisk corresponds to *p*≤0.05 and double asterisks stands for *p*≤0.001. *CDH1* expression, an epithelial marker, was not significantly altered during EMT/MET induction; *Ocln* expression, an epithelial marker, was significantly decreased in M cells (in comparison to E cells) and recovered in RE cells (in comparison to M cells); *Vim* expression, a mesenchymal marker, was significantly increased in M cells (in comparison to E cells) and decreased in RE cells (in comparison to M cells) and; *Zeb2* expression, a classical EMT inducer, was significantly increased in M cells in comparison with E cells, supporting EMT occurrence.

The *EpH-4* cell line derives from a normal mammary gland of a mid-pregnant BALB/c mouse that underwent spontaneously immortalization [24]. To induce EMT, we have selected a previously described approach, using a pleiotropic cytokine, in particular the transforming growth factor-β1 (TGF-β1) [25]. TGF-β1 supply during 7 days efficiently induced EMT in original epithelial *EpH-4* cells (E), and thus mesenchymal *EpH-4* cells (M) were generated (Figure 1A). Removal of TGF-β1 from the culture medium led to phenotypic reversion from mesenchyme back to epithelia (RE), after four days. The MET process was terminated at day four after which bright field microscopy revealed a widespread recovery of the epithelial-like phenotype (Figure 1A). Three distinct time-points were created for biological material collection (DNA, RNA, protein and fixed cell monolayers) corresponding to *EpH-4* cells with distinct phenotypical features (E, M and RE). To understand whether the observed phenotypic alterations were due to EMT and MET induction, we next analysed the mRNA expression by qRT-PCR of several classical epithelial (*CDH1* and *Ocln*) and mesenchymal (*Vim*) markers, as well as a well-known EMT inducer (*Zeb2*). We have observed a slight decrease in *CDH1* mRNA expression upon EMT, which was recovered with MET (Figure 1B). *Ocln*, encoding occludin, was shown to decrease with EMT (E *vs.* M *p* = 1.34E-05), being recovered in MET (M *vs.* RE *p* = 4.07E-02). The opposite variation was observed for *Vim* encoding vimentin (E *vs.* M *p* = 1.60E-02 and M *vs.* RE *p* = 5.20E-02) (Figure 1B). *Zeb2* mRNA showed a significant increase in EMT (E *vs.* M *p* = 1.91E-02) and no significant variation in MET (Figure 1B).

**Figure 2:**
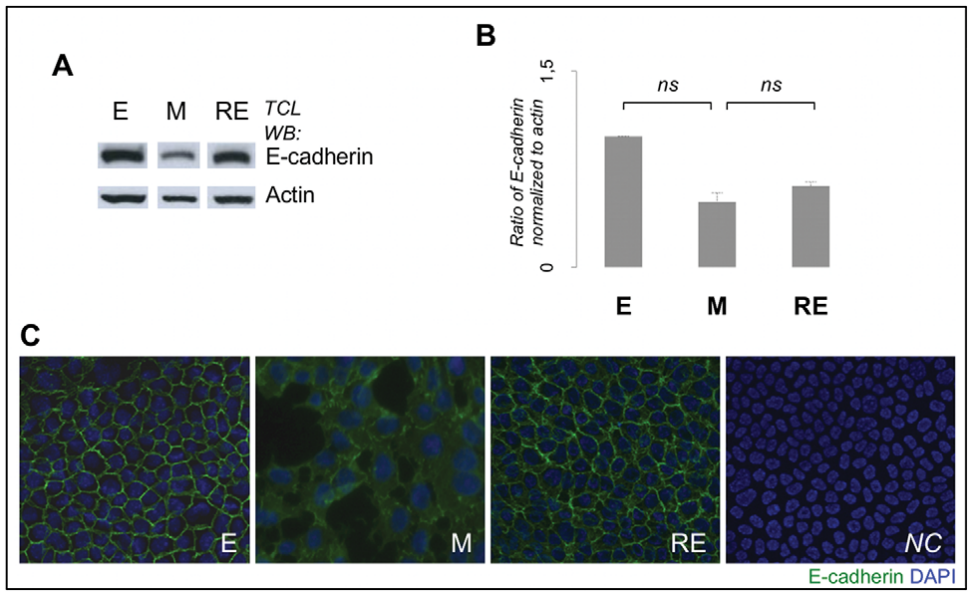
EMT/MET model validation. Panel A shows the Western blot for E-cadherin. Panel B illustrates the quantification of E-cadherin across the EMT/MET induction (*n* = 3 biological replicas). Data was normalized for E cells. Results are described as mean±standard error mean of 3 biological replicas. No significant differences were observed concerning E-cadherin expression (*ns* stands for non-significant, *p*>0.05). Panels A and B show that E-cadherin expression is decreased in M cells (in comparison to E cells) and partially recovered in RE cells (in comparison to M cells). Panel C represents the immunofluorescence for E-cadherin during EMT/MET induction (200×). *NC* stands for negative control (no E-cadherin antibody used). Panel C illustrates the variation of E-cadherin localization during the EMT/MET induction: E cells display the classical E-cadherin expression at the cell membrane; M cells show a decreased expression of E-cadherin which is only observed in some points of intercellular contacts and in the cytoplasm; RE cells display E-cadherin expression in the cell membrane.

Given that *CDH1* mRNA expression was only slightly decreased when comparing *EpH-4* E and M cells, we next analyzed the expression of the encoded protein, E-cadherin, by western blot. We observed that E-cadherin expression decreased with EMT and was partially recovered with MET (Figure 2A and 2B), however these variations were not statistically significant. By immunofluorescence, we observed an E-cadherin de-localization from the cell membrane (E cells) to the cytoplasm in M cells, pointing to a lack of functionality of this protein as a cell-adhesion molecule in M cells (Figure 2C). In *EpH-4* RE cells, E-cadherin was recovered to the cell membrane (Figure 2C).

Overall, our results point to the occurrence of EMT as demonstrated by morphological alterations observed by bright field microscopy; the expression of classical mesenchymal markers and the de-localization of the classical epithelial marker E-cadherin to the cytoplasm, commonly associated with its loss of function. Moreover, we observed a phenotypic reversion to an epithelial phenotype (although not complete), which was accompanied by an epithelial transcriptional program together with a recovery of E-cadherin at its classical membranous localization.

### *Mgat3* gene expression is significantly down regulated during EMT being significantly recovered in MET and is associated with *Mgat3* promoter methylation/demethylation

**Figure 3:**
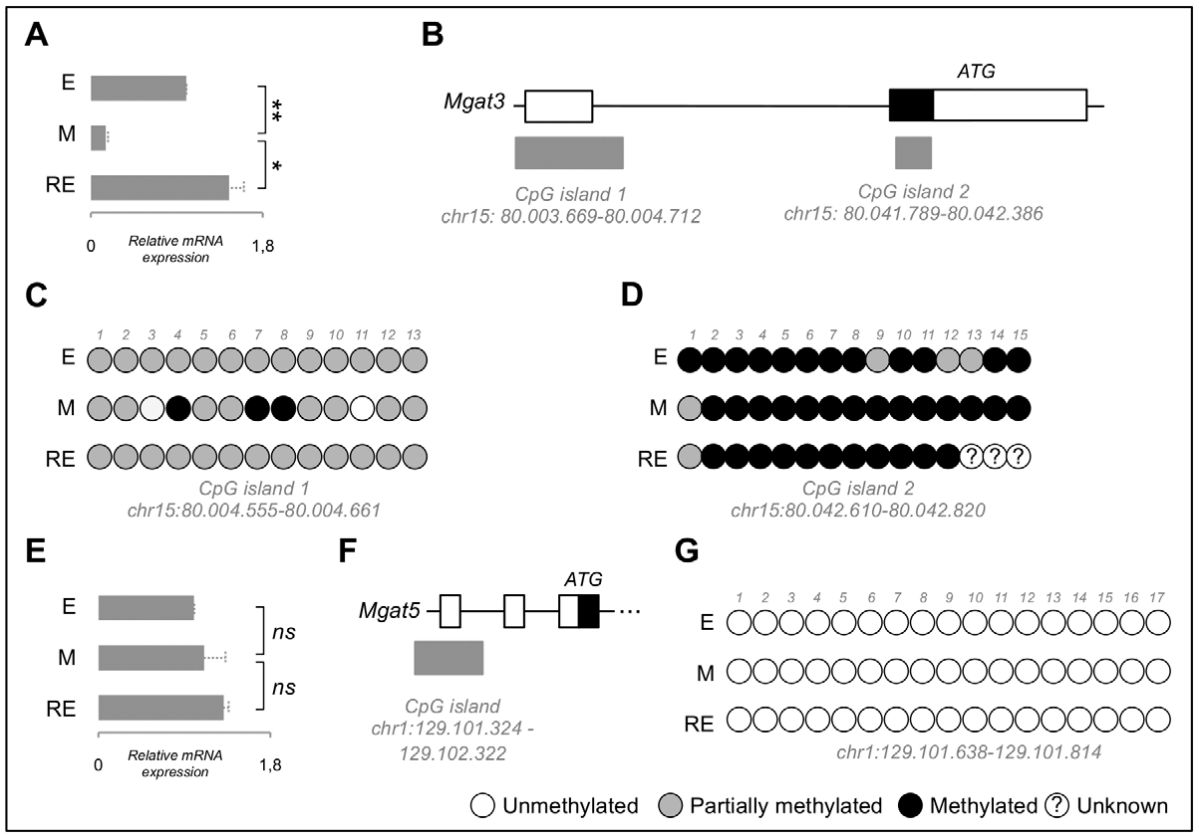
*Mgat3* and Mgat5 RNA expression and methylation status of their predicted promoter-associated CpG islands. Panel A illustrates the quantification of *Mgat3* relative mRNA expression (*n* = 3 biological replicas). Data was normalized for E cells for each biological replica. Single asterisk corresponds to p≤0.05 and double asterisks stands for p≤0.001. Results are described as mean ± standard error mean of three biological replicas. Panel A shows that *Mgat3* expression was significantly decreased in M cells (in comparison to E cells) and recovered in RE cells (in comparison to M cells). Schematic representation of *Mgat3* genomic locus is represented in panel B. White squares correspond to exonic untranslated regions and black squares to exonic translated regions. Black line stands for intronic regions. Grey squares represent the position of the bioinformatically predicted CpG islands (classified as 1 and 2). Panel C and D show the schematic representation of the methylation status of several CpG dinucleotides evaluated within CpG islands 1 (C) and 2 (D) of *Mgat3* across the EMT/MET experiment (E, M and RE). White circles correspond to unmethylated CpGs, grey circles correspond to partially methylated CpGs, black circles correspond to methylated CpGs, white circles with a question mark correspond to unknown methylation status. Panel C shows methylation pattern alterations across several CpG sites within *Mgat3*'s CpG island 1 in E, M and RE cells. Panel E illustrates the quantification of Mgat5 relative mRNA expression (*n* = 3 biological replicas). Same legend as in A applies. No significant variation of Mgat5 RNA expression was observed during EMT/MET. Schematic representation of part of the Mgat5 genomic locus is represented in panel F. Same legend as in panel B applies. Schematic representation of the methylation status of several CpG dinucleotides evaluated within the annotated Mgat5 CpG island is represented in panel G. Same legend as in panels C and D applies. The results showed no variation of the methylation status of Mgat5 promoter during EMT/MET.

*Mgat3* mRNA expression was evaluated by qRT-PCR during EMT/MET. We observed a significant decrease in *Mgat3* mRNA expression upon EMT (*p* = 8.10E-04), followed by a full recovery upon MET (*p* = 1.25E-02) (Figure 3A). To understand whether this down-regulation could be related with a classical inactivating mechanism, we have analysed the methylation status of *Mgat3* promoter region.

Due to the lack of a described mouse *Mgat3* promoter in the literature, we pursued the classical approach of analysing the bioinformatically predicted *CpG* islands within the gene locus, given the known association between *CpG* islands and promoter regions [26] (Figure 3B). We observed that the methylation pattern of *Mgat3 CpG* island 1 was significantly altered with EMT (Figure 3C): 1) three *CpG* sites became completely methylated (*CpG* sites 4, 7 and 8); 2) two *CpG* sites became completely demethylated (*CpG* sites 3 and 11). We also observed that the *CpG* island 1 methylation pattern observed for *EpH-4* cells E and RE reproduced the *Mgat3* mRNA expression levels (Figure 3A and C). Concerning *CpG* island 2, no significant alterations were detected during the EMT/MET. Overall this *CpG* island was permanently methylated throughout the experiment (Figure 3D). To confirm that methylation of *Mgat3* promoter was a specific feature of this gene, potentially leading to its expression downregulation, we also analysed the methylation status of the promoter of *Mgat5*, encoding the GnT-V glycosyltransferase, for which RNA expression did not vary during the EMT/MET experiment (Figure 3E and F). The methylation status of *Mgat5* did not change along EMT/MET (Figure 3G).

These results highlighted a novel regulatory mechanism controlling *Mgat3* expression involving *CpG* island promoter methylation associated with EMT and MET.

### Evaluation of the levels of expression and cellular localization of bisecting GlcNAc structures during EMT/MET

**Figure 4:**
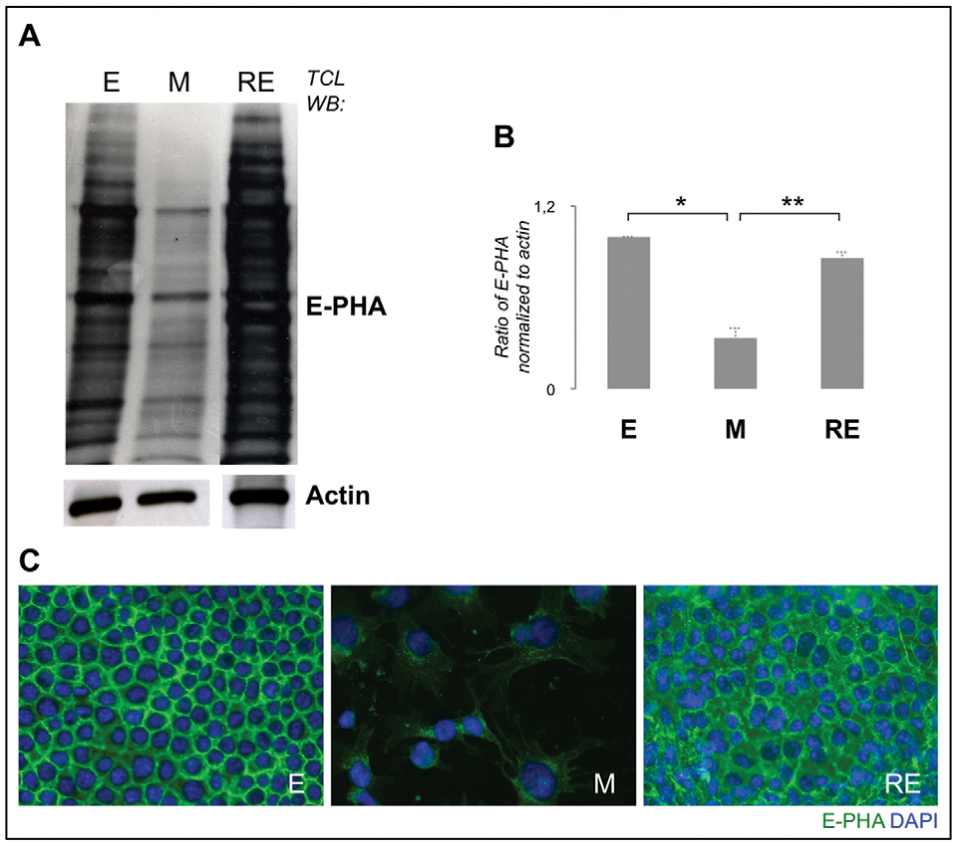
Expression levels and cellular localization of the product of GnT-III enzyme during EMT/MET induction. Panel A shows the lectin blot analysis using E-PHA lectin, showing the total expression levels of bisecting GlcNAc structures during EMT/MET. Panel B illustrates the quantification of E-PHA lectin normalized to actin (*n* = 2 biological replicas). Single asterisk corresponds to *p*≤0.05 and double asterisks stands for *p*≤0.001. Bisecting GlcNAc structures significantly decrease when comparing E and M cells and their expression is significantly recovered in RE cells. Panel C represents the immunofluorescence for E-PHA lectin during EMT/MET induction (400×). Bisecting GlcNAc structures are preferentially localized in the cell membrane of E cells. M cells exhibit a clear decrease in expression of the bisecting GlcNAc structures that was only observed in focal areas in the perinuclear region. In RE cells, there was a significant increase in the E-PHA staining showing an increase in the expression levels of bisecting GlcNAc structures that are localized in the cell membrane and in the cytoplasm.

Taking into consideration the results at the transcriptional level of *Mgat3* gene during EMT/MET, we analyzed the product of the activity of the GnT-III enzyme, the bisecting GlcNAc structures (Figure 4). We performed E-PHA lectin blot analysis, which specifically recognizes the product of GnT-III. The results showed that the total levels of expression of bisecting GlcNAc structures significantly decrease during EMT (*p* = 3.3E-03), with E cells displaying a significant increased expression of bisecting GlcNAc structures than M cells (Figure 4A, 4B). The total levels of expression of bisecting GlcNAc structures was dramatically recovered in RE cells when compared with M cells (*p* = 8.0E-04).

The evaluation of the cellular localization of the product of GnT-III (bisecting GlcNAc structures), by performing E-PHA lectin IF, clearly showed that during EMT there was a remarkable decrease in the levels of expression of these structures, as observed in M cells, that were recovered in RE cells. In terms of cellular localization, the expression of the GnT-III product in E cells was mainly in the cell membrane with rare cytoplasmic staining; in M cells very few E-PHA lectin staining was observed restricted at the cytoplasm in a perinuclear location, and in RE cells E-PHA lectin staining was observed both at the cell membrane and cytoplasm (Figure 4C).

These results showed that high levels of expression of bisecting GlcNAc structures are associated with epithelial phenotypes (E, RE) and that these structures localize at the cell membrane only in these phenotypes.

### E-cadherin is a specific target of regulation by GnT-III mediated glycosylation during EMT/MET

**Figure 5:**
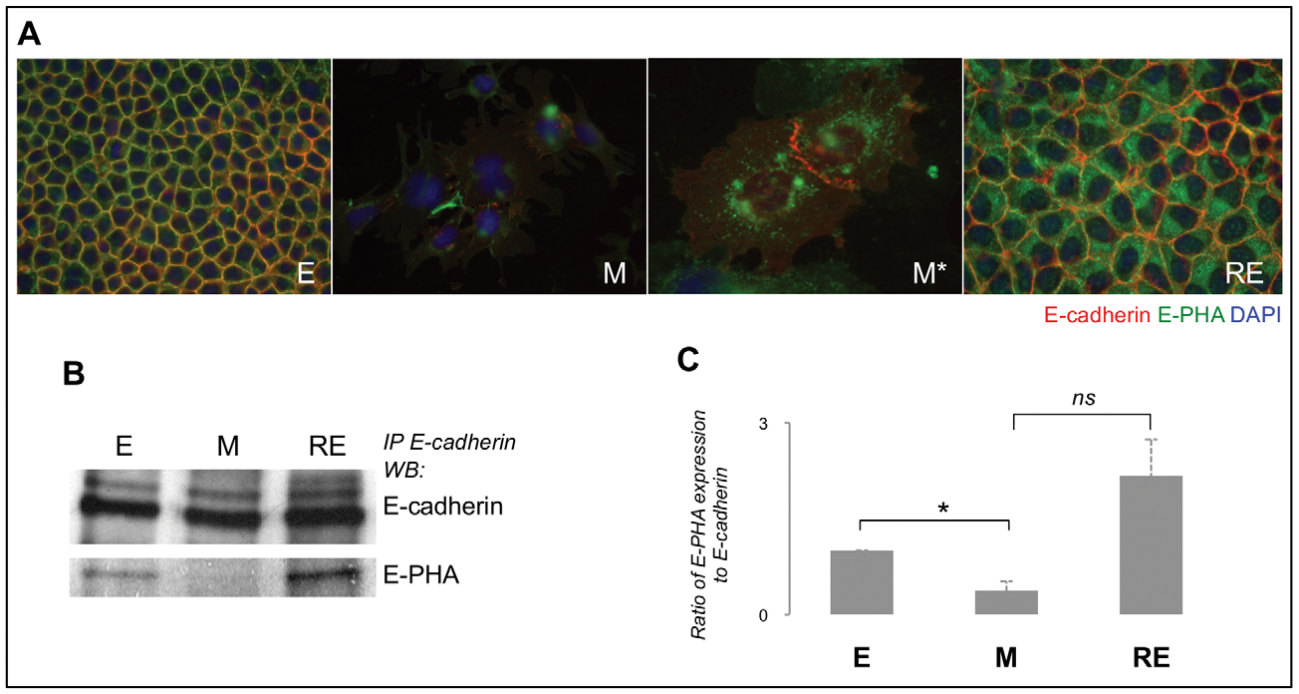
GnT-III-mediated E-cadherin glycosylation during EMT/MET. Panel A shows the co-immunofluorescence for E-cadherin and E-PHA (400× for E, M, RE and 630× for M*) illustrating that E-cadherin and bisecting GlcNAc structures co-localize in the cell membrane in E and RE. In mesenchymal cells (M and M*), it was observed a significant decrease in both the expression of E-cadherin and bisecting GlcNAc structures. M cells shows residual E-cadherin expression at the focal points of intercellular contacts (red, E-cadherin) and some green staining (E-PHA reactivity) could be observed in the perinuclear region (Golgi compartment). Immunoprecipitation of E-cadherin followed by E-PHA lectin blot is represented in panel B. Panel C represents the normalization of bisecting GlcNAc structures (E-PHA reactivity) that are modifying E-cadherin. Amounts of N-glycan structures were determined from the ratios of densities of E-PHA reactivity after normalization to E-cadherin. Results are described as mean ± standard error mean of two biological replicas. Single asterisk corresponds to *p*≤0.05 and *ns* stands for non-significant, *p*>0.05. The modification of E-cadherin with bisecting GlcNAc N-glycan structures in E, M and RE are expressed as the fold increase, compared with the E cells. Panels B and C show that E-cadherin is specifically glycosylated with bisecting GlcNAc structures in E, losing this glycoform in M and recovering again in RE.

As the expression of bisecting GlcNAc structures and E-cadherin was observed at the cell membrane in epithelial phenotypes (E and RE), we next evaluated the co-localization of both molecules by performing double-labelled immunofluorescence (Figure 5A). The results showed that in both E and RE cells there was a co-localization of bisecting GlcNAc structures (green colour) and E-cadherin (red colour) at the cell membrane. During EMT, when cells acquire a mesenchymal phenotype, the co-localization disappears and cells show few bisecting GlcNAc structures in the cytoplasm in a perinuclear position (that may correspond to the Golgi apparatus), whereas E-cadherin expression is limited only to the focal points of intercellular contact (Figure 5A).

We next examined whether the EMT/MET marker, E-cadherin, would be a target of regulation by the GnT-III glycosyltransferase (Figure 5B, 5C). For doing so, we performed E-cadherin IP followed by E-PHA lectin blot analysis to evaluate the specific modification of E-cadherin with bisecting GlcNAc structures, catalyzed by GnT-III during EMT/MET. We performed a normalization of the densities between E-PHA lectin band and the correspondent E-cadherin band in order to allow the comparison of the levels of E-cadherin glycosylation by GnT-III along EMT/MET. The results clearly demonstrated that during EMT E-cadherin suffered a significant decreased modification with bisecting GlcNAc structures of about 50% (*p* = 5.1E-02) (Figure 5C). On the contrary, during the reverted process (MET) E-cadherin glycosylation with bisecting GlcNAc structures was recovered for levels on average higher than those of the starting point (parental epithelial cells).

These results demonstrate that E-cadherin is being specifically glycosylated by GnT-III and that this post-translational modification varies significantly during EMT/MET.

## Discussion

The transition between epithelial and mesenchymal states requires alterations in cell morphology, cellular architecture, adhesion, and migration capacity [16], [17]. Current interest in these processes (EMT/MET) stem from their developmental importance and their involvement in several adult pathologies including cancer [21], therefore it is crucial to unravel the mechanisms involved in these transitions.

In the present manuscript, we describe for the first time that transcription of *Mgat3* gene, that encodes the GnT-III glycosyltransferase, significantly varies along EMT/MET and that its expression is controlled by promoter methylation. Further, we also showed that the product of GnT-III, the bisecting GlcNAc structures, specifically glycosylates and regulates E-cadherin functions.

GnT-III, has been considered a key glycosyltransferase during the N-glycan biosynthetic pathway [6], and has been also pointed as a metastases suppressor gene [11]. This role in the suppression of tumour invasion and metastases appears to be through glycosylation and regulation of a critical tumour suppressor gene, E-cadherin [12], [15], [27], as well as through regulation of Integrin-mediated cell-ECM adhesion [13]. Previously, we have described the existence of a functional feed-back loop between E-cadherin and GnT-III, where we found that E-cadherin expression regulates the transcription of *Mgat3* gene, which in turns leads to glycosylation of E-cadherin with bisecting GlcNAc structures [15], promoting its cell surface stability and function (unpublished results). The perturbation of this stable loop either by GnT-III knockdown or by increased competition with GnT-V enzymatic activity was shown to lead to alterations in cell morphology and E-cadherin subcellular localization [15]. These results pointing to a regulation of E-cadherin function through glycosylation [10], led us to postulate that *Mgat3* expression and GnT-III enzymatic product could interfere with E-cadherin during EMT/MET, a process in which changes in E-cadherin function are described to be pivotal [16], [17], [28].

**Figure 6:**
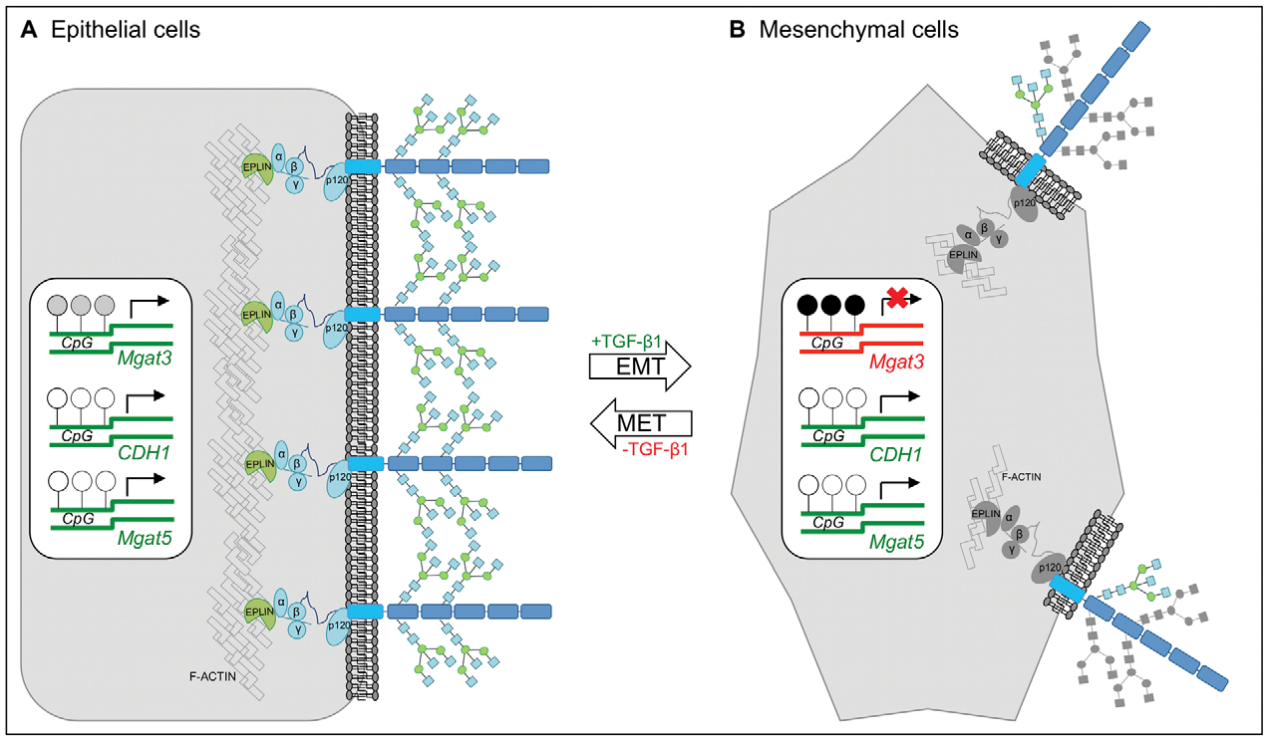
Proposed model. Panel A illustrates the following: in epithelial cells, *CDH1*, *Mgat3* and *Mgat5* are transcribed. Partial promoter methylation of *Mgat3* and no methylation of *CDH1* and *Mgat5* promoters were observed. The transcription levels of *Mgat3* generate sufficient GnT-III enzyme levels that catalyze the addition of bisecting GlcNAc structures, specifically on E-cadherin. No information is available concerning the status of the remaining molecules in the adhesion complex (catenins). Panel B illustrates the following: in mesenchymal cells, *Mgat3*'s promoter is methylated in some *CpG* sites which were associated with a significant decrease of *Mgat3* transcription. No significant changes were observed in terms of both promoter methylation status and transcription of *CDH1* and *Mgat5*. There was a significant decrease of GnT-III-mediated E-cadherin glycosylation. In reverted epithelial cells, *Mgat3*'s promoter methylation status returns to its status in original epithelial cells accompanied with a significant increase of *Mgat3* transcription, in comparison to mesenchymal cells. Concomitantly there was an increased GnT-III-mediated E-cadherin glycosylation, resembling that observed in original epithelial cells.

Using a TGF-β1-induced EMT/MET cell line model, we describe for the first time that *Mgat3* gene was significantly down regulated in mesenchymal (M) cells and was significantly recovered in cells that re-acquired an epithelial-like phenotype after removing TGF-β1 (RE cells). This suggests that *Mgat3* gene expression and the GnT-III enzymatic product may be critical to maintain the cellular epithelial and differentiated phenotype, while the sharp drop at the mesenchymal state may pinpoint the need for *Mgat3* down regulation to the acquisition of this phenotype. Moreover, we have also provided compelling evidence of increased *CpG* island methylation at the *Mgat3* promoter, underlying the down regulation of *Mgat3* expression in M cells (Figure 6).

Changes in glycogenes expression leads to alterations in the glycosyltransferases expressions levels that have major impact in the remodelling of cell surface glycosylation of glycoproteins, with consequent impact on several cellular mechanisms such as cell adhesion [5], [29]. The observed similar expression variation of the *Mgat3* gene and the product of GnT-III, the bisecting GlcNAc structures, along EMT/MET further support that, on one hand the expression of bisecting GlcNAc structures is crucial for the preservation of epithelial cell phenotype, as previously described by Iijima *et al* [27], and the absence or low levels of bisecting GlcNAc structures are needed for cells to acquire mesenchymal features.

Moreover, our current results are consistent with our previous report that MCF-7/AZ cells with *Mgat3* knockdown, displayed a remarkable modification of the cellular phenotype with disruption of the cell-cell contacts, increased lamellipodia and filopodia formation and E-cadherin internalization to the cytoplasm (mesenchymal features) [15].

Given the previously described bi-directional crosstalk between E-cadherin and GnT-III glycosylation [15], in the present study we examined the specific modification of E-cadherin with bisecting GlcNAc structures during EMT/MET. We found that, although the variation of the *CDH1*/E-cadherin during EMT/MET was not significant, E-cadherin was glycosylated with bisecting GlcNAc glycoforms and they co-localized at the membrane preferentially in E and RE cells.

Altogether, the results herein presented, lead us to propose that loss and recovery of *Mgat3* expression and GnT-III mediated glycosylation is a pivotal mechanism during EMT/MET with major consequences on E-cadherin subcellular localization. We showed a novel perspective over the regulation of E-cadherin function along EMT/MET by demonstrating that E-cadherin glycosylation with bisecting GlcNAc structures, catalysed by GnT-III enzyme, has major consequences in the regulation of its function in the EMT/MET biological processes.

These cause-consequence events, involving changes in *Mgat3* expression, GnT-III enzymatic product, E-cadherin protein glycosylation and subcellular localization are likely to uncover a mechanism controlling the transition between epithelial and mesenchymal states, therefore revealing a novel regulatory path during EMT/MET (Figure 6).

## Materials and Methods

### TGF-β-induced EMT/MET in a non-tumourigenic mammary epithelial cell line

*EpH-4* cell line [24] (classified as E for epithelial) was cultured in D-MEM/F12 *Glutamax*™ (Invitrogen, Oregon, USA) supplemented with fetal bovine serum (Lonza, Switzerland), penicillin-streptomycin (Invitrogen, Oregon, USA) and recombinant human insulin (Invitrogen, Oregon, USA). Mesenchymal cell cultures (classified as M) were obtained by supplementing the normal culture medium with transforming growth factor-β1 (TGF-β1, Sigma-Aldrich-Aldrich, Missouri, USA) during 7 days [25]. Reverted-epithelial cell cultures (classified as RE) were obtained by replacing the TGF-β1 enriched medium in mesenchymal cell cultures by normal culture medium for another 4 days.

### RNA expression quantification

RNA was extracted from 3 biological replicas of *EpH-4* cell line stages (E, M and RE) using the *mirVana miRNA Isolation Kit* (Invitrogen, Oregon, USA), according to the kit's instruction manual. Using *TaqMan Gene Expression Assays* (Applied Biosystems, California, USA), we have quantified the mRNA expression levels of *Mgat3*, *CDH1*, *Ocln*, *Zeb2*, and *Vim*. Approximately 1000 ng of total RNA were reversed transcribed to single stranded cDNA using Superscript II Reverse Transcriptase and random hexamer primers (Invitrogen, Oregon, USA). Quantitative Real-Time-PCR (qRT-PCR) was carried out in triplicates using source RNA from 3 biological replicas of the EMT/MET experiment, for the target genes *Mgat3*, *CDH1*, *Ocln*, *Zeb2*, *Vim* and for the endogenous control *GAPDH* using as probe sets Mm00447798_s1, Mm00486909_g1, Mm.PT.47.16166845, Mm.PT.47.13169136, Mm01333430_m1 and 4352932E (Applied Biosystems, California, USA and Integrated DNA Technologies, Iowa, USA) respectively and an ABI Prism 7000 Sequence Detection System. Data was analysed by the comparative 2(-ΔΔCT) method [30]. For all data comparisons, the Student's T-Test was used (two tailed, unequal variance).

### *Mgat3* and *Mgat5* methylation status analysis

DNA from 3 independent biological replicas of *EpH-4* cells (E, M and RE) was extracted using the kit *Invisorb Spin Tissue Mini Kit* following the manufacturer's instructions (STRATEC Molecular, Berlin, Germany). Approximately 300 ng of DNA from each and every sample were then subjected to complete bisulfite conversion and subsequent cleanup using the *Epitect Bisulfite Kit* following manufacturer's instructions (Qiagen, Hilden, Germany). Bisulfite treated DNA was amplified using primers flanking the *Mgat3* promoter *CpG* island (Sigma-Aldrich-Aldrich, Missouri, USA), specifically designed for bisulfite treated DNA sequences without *CpG* sites. The bisulfite PCR products were sequenced for methylation status determination. *Mgat3* promoter methylation analysis was carried out within the two *CpG* islands bioinformatically predicted to exist within *Mgat3* genomic locus [26], [31]. *Mgat5* promoter methylation analysis was carried out within the single *CpG* island bioinformatically predicted to exist within *Mgat5* genomic locus [26], [31]. The criteria used for *CpG* island prediction was as follows: 1) genomic area with ≥500 bp; 2) a percentage of GC ≥55 and; 3) the observed/expected *CpG* dinucleotides ≥0.65. Using the Ensembl database [31] and the web tool “*CpG Island Searcher*” [26] and the described criteria, two distinct *CpG* islands were predicted within/in the vicinity of *Mgat3* genomic locus: *CpG* island 1, located at chr15: 80003669–80004712; *CpG* island 2, located at chr15: 80041789–80042386. In particular, the methylation status was analysed at 13 *CpG* sites for *CpG* island 1 (chr15: 80004555–80004661) and at 15 *CpG* sites for *CpG* island 2 (chr15: 80042610–80042820). Concerning *Mgat5*, the predicted *CpG* island was located at chr1:129.101.324–129.102.322. In particular, the methylation status was analysed at 17 *CpG* sites (chr1:129.101.638–129.101.814). Only results defining the findings obtained for all biological and technical replicas are herein presented.

### Western-blot, Immunoprecipitation and Lectin Blot Analysis

*EpH-4* cells (E, M and RE) were washed with PBS and then lysed in cold PBS containing 1% TritonX-100, 1% NP40, protease inhibitor cocktail (Roche 1tablet/50 ml buffer) and phosphatase inhibitor cocktail (Sigma-Aldrich, 1∶100 dilution). Total protein was quantified using a BCA protein assay kit (Pierce).

Equal amounts of total cell protein lysates (25 µg) from each cell were subjected to 7.5% SDS-PAGE electrophoresis and then transferred to nitrocellulose membranes. After blocking with 5% nonfat milk (for western-blot) or with 5% BSA in PBS (for lectin blot), the membranes were incubated with primary antibodies against human E-cadherin (clone 36, BD Transduction Laboratories and Cell Signaling). For the lectin blot analysis, membranes were incubated with E-PHA lectin (Vector Laboratories). Next, membranes were washed four times with PBST solution, followed by incubation with horseradish peroxidase-linked secondary antibody for the E-cadherin blot analysis. For the lectin blots, the immunoreactive bands were visualized using the Vectorstain ABC kit (Vector Laboratories). Blots were then probed with anti-actin antibody (Santa Cruz Biotechnology) for loading control analysis. Results were obtained from two independent experiments using two different biological replicas.

For immunoprecipitation analysis, equal amounts of total protein from each cell lysate were precleared with 25 ml of protein G-sepharose beads (Sigma-Aldrich) for 1–2 h. After centrifugation, the supernatant was incubated overnight with 5 mg of monoclonal antibody against E-cadherin (BD Biosciences and Cell Signalling). After that, incubation with protein G-sepharose for 2 h was performed. Next, the beads were washed three times with immunoprecipitation buffer. The immune complexes were released by boiling for 5 min at 95°C in Laemmli sampling buffer and the immunoprecipitates were subjected to 7.5% SDS–PAGE; the separated proteins were transferred to a nitrocellulose membrane. The blots were then probed with the primary antibody against E-cadherin. For the bisecting GlcNAc analysis on E-cadherin IP, membranes were probed with E-PHA lectin. The results were visualized with ECL detection reagent (Amersham Biosciences). The experiment was reproduced three times using protein extract from two different biological replicas. For all data comparisons, the Student's T-Test was used (two tailed, unequal variance).

### Immunofluorescence

*EpH-4* cells at the three different stages (E, M, RE) were plated on six-well plates with coverslips on the bottom of each well. Cells from each stage (E; M; RE) were fixed with Methanol and blocked with BSA 10% in PBS.

For E-cadherin staining, cells were incubated with anti-E-cadherin monoclonal antibody (BD Biosciences and Cell Signalling) for 1 h at room temperature. After three washes with phosphate-buffered saline (PBS), cells were incubated with Alexa Fluor 488 anti-mouse secondary antibody (Invitrogen) for 1 h at room temperature.

For E-PHA staining and in the same way, *EpH-4* E, M and RE cells were incubated for 1 h at room temperature with biotinylated *Phaseolus vulgaris erythroagglutinin* (E-PHA, which binds to bisecting GlcNAc structures, product of GnT-III) lectin (Vector Laboratories). Following three washes with PBS, the cells were incubated with streptavidin-FITC for 1 h at room temperature.

For E-cadherin and E-PHA double-staining a double-labeled immunofluorescence, *EpH-4* E, M and RE cells were incubated with anti-E-cadherin monoclonal antibody (BD Biosciences and Cell Signalling) followed by Alexa Fluor 594 anti-mouse secondary antibody. Then cells were incubated with E-PHA biotinylated lectin followed by streptavidin-FITC incubation.

The nuclear staining for all the immunofluorescence experiments was performed and images were then visualized in a fluorescence microscope (Zeiss).

Each experiment was reproduced two times using protein extract from two different biological replicas.

## References

[1] Apweiler R, Hermjakob H, Sharon N (1999). On the frequency of protein glycosylation, as deduced from analysis of the SWISS-PROT database.. Biochim Biophys Acta.

[2] Dennis JW, Granovsky M, Warren CE (1999). Protein glycosylation in development and disease.. Bioessays.

[3] Fuster MM, Esko JD (2005). The sweet and sour of cancer: glycans as novel therapeutic targets.. Nat Rev Cancer.

[4] Ohtsubo K, Marth JD (2006). Glycosylation in cellular mechanisms of health and disease.. Cell.

[5] Taniguchi N, Ekuni A, Ko JH, Miyoshi E, Ikeda Y (2001). A glycomic approach to the identification and characterization of glycoprotein function in cells transfected with glycosyltransferase genes.. Proteomics.

[6] Stanley P (2002). Biological consequences of overexpressing or eliminating N-acetylglucosaminyltransferase-TIII in the mouse.. Biochim Biophys Acta.

[7] Zhao Y, Nakagawa T, Itoh S, Inamori K, Isaji T (2006). N-acetylglucosaminyltransferase III antagonizes the effect of N-acetylglucosaminyltransferase V on alpha3beta1 integrin-mediated cell migration.. J Biol Chem.

[8] Taniguchi N, Miyoshi E, Ko JH, Ikeda Y, Ihara Y (1999). Implication of N-acetylglucosaminyltransferases III and V in cancer: gene regulation and signaling mechanism.. Biochim Biophys Acta.

[9] Gu J, Taniguchi N (2004). Regulation of integrin functions by N-glycans.. Glycoconj J.

[10] Pinho SS, Seruca R, Gartner F, Yamaguchi Y, Gu J (2011). Modulation of E-cadherin function and dysfunction by N-glycosylation.. Cell Mol Life Sci.

[11] Yoshimura M, Nishikawa A, Ihara Y, Taniguchi S, Taniguchi N (1995). Suppression of lung metastasis of B16 mouse melanoma by N-acetylglucosaminyltransferase III gene transfection.. Proc Natl Acad Sci U S A.

[12] Yoshimura M, Ihara Y, Matsuzawa Y, Taniguchi N (1996). Aberrant glycosylation of E-cadherin enhances cell-cell binding to suppress metastasis.. J Biol Chem.

[13] Isaji T, Gu J, Nishiuchi R, Zhao Y, Takahashi M (2004). Introduction of bisecting GlcNAc into integrin alpha5beta1 reduces ligand binding and down-regulates cell adhesion and cell migration.. J Biol Chem.

[14] Song Y, Aglipay JA, Bernstein JD, Goswami S, Stanley P (2010). The bisecting GlcNAc on N-glycans inhibits growth factor signaling and retards mammary tumor progression.. Cancer Res.

[15] Pinho SS, Reis CA, Paredes J, Magalhaes AM, Ferreira AC (2009). The role of N-acetylglucosaminyltransferase III and V in the post-transcriptional modifications of E-cadherin.. Hum Mol Genet.

[16] Thiery JP (2002). Epithelial-mesenchymal transitions in tumour progression.. Nat Rev Cancer.

[17] Thiery JP, Sleeman JP (2006). Complex networks orchestrate epithelial-mesenchymal transitions.. Nat Rev Mol Cell Biol.

[18] Christiansen JJ, Rajasekaran AK (2006). Reassessing epithelial to mesenchymal transition as a prerequisite for carcinoma invasion and metastasis.. Cancer Res.

[19] Zeisberg M, Neilson EG (2009). Biomarkers for epithelial-mesenchymal transitions.. J Clin Invest.

[20] Hanahan D, Weinberg RA (2011). Hallmarks of cancer: the next generation.. Cell.

[21] Lee JM, Dedhar S, Kalluri R, Thompson EW (2006). The epithelial-mesenchymal transition: new insights in signaling, development, and disease.. J Cell Biol.

[22] Terao M, Ishikawa A, Nakahara S, Kimura A, Kato A (2011). Enhanced epithelial-mesenchymal transition-like phenotype in N-acetylglucosaminyltransferase V transgenic mouse skin promotes wound healing.. J Biol Chem.

[23] Pinho SS, Osorio H, Nita-Lazar M, Gomes J, Lopes C (2009). Role of E-cadherin N-glycosylation profile in a mammary tumor model.. Biochem Biophys Res Commun.

[24] Montesano R, Soriano J, Fialka I, Orci L (1998). Isolation of EpH4 mammary epithelial cell subpopulations which differ in their morphogenetic properties.. In Vitro Cell Dev Biol Anim.

[25] Robson E, Khaled W, Abell K, Watson C (2006). Epithelial-to-mesenchymal transition confers resistance to apoptosis in three murine mammary epithelial cell lines.. Differentiation.

[26] Takai D, Jones PA (2003).

[27] Iijima J, Zhao Y, Isaji T, Kameyama A, Nakaya S (2006). Cell-cell interaction-dependent regulation of N-acetylglucosaminyltransferase III and the bisected N-glycans in GE11 epithelial cells. Involvement of E-cadherin-mediated cell adhesion.. J Biol Chem.

[28] Gravdal K, Halvorsen OJ, Haukaas SA, Akslen LA (2007). A switch from E-cadherin to N-cadherin expression indicates epithelial to mesenchymal transition and is of strong and independent importance for the progress of prostate cancer.. Clin Cancer Res.

[29] Taniguchi N, Yoshimura M, Miyoshi E, Ihara Y, Nishikawa A (1998). Gene expression and regulation of N-acetylglucosaminyltransferases III and V in cancer tissues.. Adv Enzyme Regul.

[30] Livaka K, Schmittgenb T (2001). Analysis of relative gene expression data using real-time quantitative PCR and the 2(-Delta Delta C(T)) Method.. Methods.

[31] Flicek P, Amode MR, Barrell D, Beal K, Brent S (2011). Ensembl 2011.. Nucleic Acids Res.

